# Causal effects of maternal circulating amino acids on offspring birthweight: a Mendelian randomisation study

**DOI:** 10.1016/j.ebiom.2023.104441

**Published:** 2023-01-23

**Authors:** Jian Zhao, Isobel D. Stewart, Denis Baird, Dan Mason, John Wright, Jie Zheng, Tom R. Gaunt, David M. Evans, Rachel M. Freathy, Claudia Langenberg, Nicole M. Warrington, Deborah A. Lawlor, Maria Carolina Borges, MR-PREG Consortium

**Affiliations:** a MRC Integrative Epidemiology Unit, University of Bristol, Bristol, UK; b Bristol NIHR Biomedical Research Centre, Bristol, UK; c Population Health Sciences, Bristol Medical School, University of Bristol, Bristol, UK; d The Ministry of Education and Shanghai Key Laboratory of Children's Environmental Health, Institute of Early Life Health, Xinhua Hospital, Shanghai Jiao Tong University School of Medicine, Shanghai, China; e Department of Maternal and Child Health, School of Public Health, Shanghai Jiao Tong University, Shanghai, China; f MRC Epidemiology Unit, University of Cambridge, Cambridge, UK; g Bradford Institute for Health Research, Bradford Teaching Hospitals National Health Service Foundation Trust, Bradford, UK; h Department of Endocrine and Metabolic Diseases, Shanghai Institute of Endocrine and Metabolic Diseases, Ruijin Hospital, Shanghai Jiao Tong University School of Medicine, Shanghai, China; i Shanghai National Clinical Research Center for Metabolic Diseases, Key Laboratory for Endocrine and Metabolic Diseases of the National Health Commission of the PR China, Shanghai Key Laboratory for Endocrine Tumor, State Key Laboratory of Medical Genomics, Ruijin Hospital, Shanghai Jiao Tong University School of Medicine, Shanghai, China; j University of Queensland Diamantina Institute, University of Queensland, Brisbane, QLD, Australia; k Institute for Molecular Bioscience, The University of Queensland, Brisbane, QLD, Australia; l Institute of Biomedical and Clinical Science, College of Medicine and Health, University of Exeter, Exeter, UK; m Health Data Research UK Cambridge, Wellcome Genome Campus and University of Cambridge, Cambridge, UK; n Computational Medicine, Berlin Institute of Health (BIH), Charité University Medicine, Berlin, Germany; o K.G. Jebsen Center for Genetic Epidemiology, Department of Public Health and Nursing, NTNU, Norwegian University of Science and Technology, Trondheim, Norway

**Keywords:** Amino acids, Birthweight, GWAS, Mendelian randomisation, Causal effect

## Abstract

**Background:**

Amino acids are key to protein synthesis, energy metabolism, cell signaling and gene expression; however, the contribution of specific maternal amino acids to fetal growth is unclear.

**Methods:**

We explored the effect of maternal circulating amino acids on fetal growth, proxied by birthweight, using two-sample Mendelian randomisation (MR) and summary data from a genome-wide association study (GWAS) of serum amino acids levels (sample 1, n = 86,507) and a maternal GWAS of offspring birthweight in UK Biobank and Early Growth Genetics Consortium, adjusting for fetal genotype effects (sample 2, n = 406,063 with maternal and/or fetal genotype effect estimates). A total of 106 independent single nucleotide polymorphisms robustly associated with 19 amino acids (*p* < 4.9 × 10^−10^) were used as genetic instrumental variables (IV). Wald ratio and inverse variance weighted methods were used in MR main analysis. A series of sensitivity analyses were performed to explore IV assumption violations.

**Findings:**

Our results provide evidence that maternal circulating glutamine (59 g offspring birthweight increase per standard deviation increase in maternal amino acid level, 95% CI: 7, 110) and serine (27 g, 95% CI: 9, 46) raise, while leucine (−59 g, 95% CI: −106, −11) and phenylalanine (−25 g, 95% CI: −47, −4) lower offspring birthweight. These findings are supported by sensitivity analyses.

**Interpretation:**

Our findings strengthen evidence for key roles of maternal circulating amino acids during pregnancy in healthy fetal growth.

**Funding:**

A full list of funding bodies that contributed to this study can be found under Acknowledgments.

**Research in context**

**Evidence before this study**

Amino acids are essential for healthy fetal growth, however, up-to-date evidence on the contribution of specific amino acids to fetal growth is still limited due to sparce human studies conducted previously and inconsistent conclusions from small studies. The inconsistent findings from previous observational studies might be due to risk of bias suffered from confounding bias (e.g., confounded by adiposity related traits in previous studies). Thus, the causal relationship between maternal circulating amino acids during pregnancy and offspring birthweight remains unclear.

**Added value of this study**

Using two-sample Mendelian randomisation analysis, we examined the causal effect of maternal circulating amino acids during pregnancy on offspring birthweight based on summary level data from the largest genome-wide association study (GWAS) of serum amino acids levels measured by high-throughput platforms (86,507 individuals) and a maternal GWAS of offspring birthweight (406,063 individuals with maternal and/or fetal genotype effect estimates) in UK Biobank and Early Growth Genetics Consortium. We found several potentially biologically meaningful maternal circulating amino acids having causal effects on offspring birthweight.

**Implications of all the available evidence**

Our findings indicate that maternal genetically predicted levels of glutamine and serine increase offspring birthweight, and leucine and phenylalanine appear to reduce offspring birthweight. Future randomized controlled trials are warranted to identify potential intervention opportunity (e.g., amino acids supplementation during pregnancy) to optimize healthy fetal growth.

## Introduction

Healthy fetal growth and development are essential for survival, short-term, and potentially longer-term health.^1^, ^2^, ^3^ Amino acids are essential for the synthesis of protein and numerous other molecules, as well as for the modulation of multiple cell signalling pathways. It has been estimated that amino acids need to be supplied at rates between 10 and 60 g/day per kg fetus for adequate fetal growth.^4^ In vivo human studies highlight complex interactions between maternal, placental and fetal mechanisms in how different amino acids are delivered to the fetus.^5^, ^6^, ^7^ However, evidence from human studies on how maternal amino acids influence fetal growth and development is scarce, with inconsistent conclusions from small studies of amino acids supplementation in pregnancies at risk of fetal growth restriction.^8^, ^9^, ^10^, ^11^

Mendelian randomisation (MR) is an approach where genetic variants, robustly associated with a modifiable exposure, are used as instrumental variables to infer the causal relationship between the exposure and an outcome of interest.^12^^,^^13^ Maternal genetic variants, normally single nucleotide polymorphisms (SNPs), have been increasingly used as instrumental variables to examine the causal relationships between genetically influenced intrauterine exposures and offspring birthweight in MR analysis.^14^, ^15^, ^16^, ^17^ These have confirmed the causal effect of maternal smoking during pregnancy on slower fetal growth as assessed by repeated ultrasound scan and lower birthweight,^18^^,^^19^ and of pre-pregnancy body mass index and higher fasting glucose on higher birthweight.^17^ MR is less likely to be biased by the socioeconomic, environmental, behavioural and health factors that confound conventional multivariable analyses, although it is subject to other sources of bias such as weak instrument bias and bias from unbalanced horizontal pleiotropy (discussed below).^20^, ^21^, ^22^, ^23^ It may also provide a long-term (possibly across the whole life course) assessment of between person differences in an exposure. In relation to this study, it could establish evidence of the effect of maternal circulating amino acids during pregnancy on fetal growth (as indicated by birthweight).

The aim of this study was to use two-sample MR to estimate the potential causal effect of maternal serum levels of the 20 established amino acids on offspring birthweight in up to 406,063 individuals with maternal and/or fetal genotype effect estimates.

## Methods

We selected genetic variants strongly associated with 20 different circulating amino acids from the largest genome-wide association studies (GWAS) available (N up to 86,507),^24^ which were validated in samples of pregnant women (N = 2,966) from the Born in Bradford (BiB) study—the only study with maternal pregnancy circulating amino acids and genome wide data that we could identify^25^—and women only (N = 4,407) using data from the Fenland study included in the main GWAS.^24^ We used these genetic variants as instruments to examine the effects of maternal circulating amino acids during pregnancy on offspring birthweight in a two-sample summary data MR framework.^22^^,^^26^

### Data sources

#### Sample 1: estimates for the association between genetic variants and amino acids

Summary data for the association between genetic variants and amino acids were retrieved from a recently conducted cross-platform GWAS of 174 metabolites that included 20 amino acids and up to 86,507 adult women and men (for individual metabolites sample sizes varied from 8,569 to 86,507).^24^ Genome-wide association analyses of up to 174 plasma metabolites were undertaken in the following cohorts of UK adults.

• Fenland study (N = 9,363 adults) in which 174 metabolites were measured using mass spectrometry (MS, Biocrates p180 kit).
• EPIC-Norfolk (N = 5,841) in which metabolites were measured using MS (Metabolon Discovery HD4).
• INTERVAL study in which metabolites were measured using MS (Metabolon Discovery HD4 platform, N = 8,455) and proton nuclear magnetic resonance (^1^H NMR, Nightingale, N = 40,905).

Given that two genotyping arrays (Affymetrix Axiom and Affymetrix SNP5.0) were used in the Fenland study, genome-wide association meta-analysis based on the two chips were first undertaken in the Fenland study and then results were meta-analysed with genome-wide association results in EPIC-Norfolk and INTERVAL for metabolites that matched those measured in the Fenland Biocrates platform. These results were further meta-analysed with publicly available GWAS summary data from two studies.

• GWAS meta-analysis of 123 metabolites measured using NMR spectroscopy on up to 24,925 individuals from 14 cohorts in Europe by Kettunen et al.^27^
• GWAS meta-analysis of more than 400 metabolites measured using the MS Metabolon platform on up to 7,824 individuals from two European population studies (KORA and TwinsUK) by Shin et al.^28^

For each metabolite, a meta-analysis of z-scores was performed based on the above summary data using METAL. Quality control was performed after meta-analysis with excluding variants with minor allele frequency (MAF) below 0.5% and not captured by at least half of the participating studies or sample size for each metabolite measured.^24^

A total of 20 amino acids (alanine, arginine, asparagine, aspartate, cysteine, glutamate, glutamine, glycine, histidine, isoleucine, leucine, lysine, methionine, phenylalanine, proline, serine, threonine, tryptophan, tyrosine and valine) were included in the meta-analysis. We calculated the SNP effect and standard error based on the z-score, sample size and MAF reported in the abovementioned z-score based meta-analysis results using the method introduced in ref.^29^

#### Sample 2: estimates for the association between maternal genetic variants and offspring birthweight

Summary data for the association between genetic variants and birthweight were extracted from the most recent GWAS of birthweight which included 297,356 individuals who reported their own birthweight and 210,248 women who reported their offspring's birthweight were combined into analysis (total n = 406,063).^30^ Participants with gestational age less than 37 completed weeks (where known) or birthweight less than 2.5 kg or greater than 4.5 kg (in UK Biobank) were excluded. Associations with birthweight were in standard deviation (SD) units in this GWAS and we multiplied these by 484 (the median SD of birthweight in the 18 prospective cohorts included in this GWAS) to obtain results in grams, which are easier to interpret.

In this study, we were primarily interested in using maternal genetic variants to test the effect of maternal circulating amino acids on offspring birthweight. One challenge of utilising MR to test causal intrauterine effects on offspring outcomes, such as birthweight, is the correlation between maternal and offspring genotypes. If the offspring genetic variants affect the offspring outcome, there could be violation of the exclusion restriction assumption. The GWAS by Warrington et al. used a newly developed structural equation modelling (SEM) approach to partition maternal and fetal genetic effects on birthweight.^30^^,^^31^ In this study, we used the summary data from the association of maternal genetic variants on offspring birthweight, adjusted for offspring genetic effects, as provided by the weighted linear model adjusted (WLM-adjusted) analyses, an approximation of the SEM approach.

### Genetic instrumental variable selection and harmonisation with birthweight GWAS results

A total of 112 SNPs were identified as independent signals (LD-clumping was performed using r^2^ < 0.05 and ≥ 1 Mb on each side of sentinel SNP) and metabolome wide-adjusted GWAS significant with at least one of the 20 amino acids in the metabolites GWAS. In agreement with the cross-platform GWAS, we used a *p-*value <4.9 × 10^−10^ to select SNPs strongly associated with one or more amino acids. This *p*-value threshold, which considers multiple testing resulting both from the genome-wide analyses and multiple phenotypes tested, was calculated as the conventional GWAS *p*-value threshold divided by the number of principal components explaining 95% of the variation in the 174 metabolites in the Fenland study (i.e., metabolome-wide adjusted GWAS *p*-value = 5 × 10^−8^/102 = 4.9 × 10^−10^).

To ensure statistical independence across SNPs instrumenting for each amino acid, we conducted a more stringent LD clumping with a cut-off of r^2^ < 0.01 within a 10,000 kb window, by using a 1000 Genomes European reference panel. As a result, 110 of the 112 SNPs passed the filtering and were left to be used as instruments for at least one of the 20 amino acids.

We estimated the pair-wise correlations across amino acids for the selected genetic variants to assess the potential genetic correlation between amino acids. To do that, we used the list of the above 110 selected SNPs to extract SNP-amino acid effect estimates for amino acids present in the summary data from previously published GWAS,^27^^,^^28^ where 8 amino acid concentrations were measured via NMR spectroscopy and 7 additional amino acids were measured via MS Metabolon in the two GWAS, respectively. We then calculated and visualised the pair-wise correlation coefficients between amino acids separately for different amino acid measurement platforms (i.e., NMR and MS Metabolon).

For the 110 selected SNPs, we searched for SNP-birthweight association summary data in the GWAS of maternal genetic effects on offspring birthweight. Five SNPs (rs8061221, rs4253272, rs1065853, rs72661853 and rs142714816) were absent from the birthweight GWAS, for four of which we could identify proxy SNPs (rs7187819, rs4253282, rs7412, rs112748538) in high linkage disequilibrium (LD) (details can be found in Table S1). No proxy SNP could be found for rs142714816, which was excluded from further analysis (Table S1).

We harmonised the SNP-exposure and SNP-outcome association data using “harmonise_data” function of the TwoSampleMR R package.^32^ During the data harmonisation, 3 palindromic SNPs (rs28601761, rs2422358 and rs1935), with MAF of 0.42, 0.44 and 0.48, were removed because we could not unambiguously harmonise them. In the end, 106 SNPs were selected to be used as genetic instruments for the 19 amino acids (no available genetic instruments for cysteine after data harmonisation). There was considerable overlap in SNP-amino acid associations, with 6 amino acids associated with rs715, 7 amino acids associated with rs1260326, and 2 amino acids associated with other SNPs respectively as shown in Fig. S1.

### Statistical analyses

For the main MR analysis, we used Wald ratios (for glutamate and methionine because only one SNP was available for each of the two amino acids), and multiplicative random-effect inverse variance weighted (IVW) approach^33^^,^^34^ for all other amino acids, to estimate the causal effect of maternal circulating amino acids on offspring birthweight. Both of these methods assume that all instruments are valid (e.g., no horizontal pleiotropy), even though IVW could still produce unbiased estimates in certain scenarios of IV assumptions violations (i.e., balanced horizontal pleiotropy). In this study, we focus on effect size and precision, and interpret *p-*values as evidence strength indicators.^35^^,^^36^ All statistical analyses were performed using R (version 4.0.2).

### MR sensitivity analyses

We conducted a series of sensitivity analysis to assess the plausibility of the core Mendelian randomization assumptions (i.e., that the SNPs are valid and strong instruments for testing the effect of maternal circulating amino acids on offspring birthweight).

#### Instrument validity

Where more than one genetic instrument was available for a particular amino acid, we checked for the presence of outlier SNPs using leave-one-out analyses. For amino acids which have five or more SNPs as instrumental variables, we calculated Cook's distance to ascertain whether any individual SNP had a disproportionate level of influence on the analysis results where a cut-off of 4/the number of SNPs was used. We used Cochrane's Q statistic to examine the heterogeneity between SNP-specific causal estimates. The presence of outlier SNPs and substantial heterogeneity across SNPs could be indicative of horizontal pleiotropy. Where five or more genetic instruments were available for a particular amino acid, we performed MR methods that are more robust to horizontal pleiotropy, such as weighted median^20^ and MR-Egger regression.^37^ The weighted median method assumes that more than 50% of the weight in the analyses come from valid instruments and is more robust to the effects of outliers. Unlike IVW, MR-Egger does not constrain the regression slope between the SNP-amino acids and SNP-birthweight associations to go through zero. This means that the slope (MR estimate of the causal effect) is expected to be corrected in the presence of unbalanced horizontal pleiotropy as long as the INSIDE (‘Instrument Strength Independent of Direct Effect’) assumption holds. A non-zero intercept from MR-Egger indicates unbalanced pleiotropy. We compared the consistency of results across these MR methods with our main analysis results.

Furthermore, we performed a ‘conservative MR analysis’,^38^ in which we selected only SNPs mapping to genes involved in amino acids metabolism pathways (e.g., amino acids biosynthesis or degradation). SNPs regulating the expression/function of these genes are more likely to be credible instruments for Mendelian randomization analysis of circulating amino acids in comparison to SNPs with an unknown role in gene regulation. We prioritised SNPs for the conservative MR analyses based on results reported in the metabolites GWAS work,^24^ in which they used two approaches (a hypothesis-free genetic approach and a biological knowledge-based approach) to prioritise likely causal genes for the observed genetic associations with metabolites (details can be found in^24^). In terms of amino acids that are the focus of our study, we further looked up the list of these prioritised genes associated with amino acids in Pathway Commons database,^39^ to confirm whether the functions of these genes are directly involved in the regulation of amino acids metabolism. One main selection criterion was used: a corresponding amino acid metabolism pathway can be identified in at least one data source among the many curated in that database, which reflects that the prioritized gene of a specific amino acid is directly involved in that amino acid metabolism. For those prioritised genes which were confirmed on their functional roles in the corresponding amino acids metabolism pathways, we included them in the either biologically or genetically conservative SNPs set to perform MR sensitivity analysis using Wald ratios method or multiplicative random-effect IVW method as appropriate. The results were compared with MR main analysis results.

Finally, at the request of reviewers we explored whether there might be bias due to specific horizontal pleiotropy from body mass index (BMI) or smoking (i.e., genetic variants instrumenting for amino acids influence BMI/smoking which in turn affect birthweight, independently of the specific amino acid). We examined the causal effect of each amino acid on birth weight, adjusting for maternal BMI and lifetime smoking in multivariable MR (MVMR) analysis separately.^40^ Summary genome-wide data on maternal BMI were extracted from GWASs on BMI of 171,977 women of European ancestry conducted by the Genetic Investigation of ANthropometric Traits (GIANT) consortium.^41^ Summary genome-wide data on lifetime smoking were extracted from 462,690 individuals of European ancestry who participated in the UK Biobank study.^42^ A total of 37 SNPs instrumenting for maternal BMI and 126 SNPs instrumenting for lifetime smoking were obtained. Genetic variants instrumenting for amino acids, BMI and lifetime smoking were harmonised with each other and with those for birthweight, where an additional round of LD clumping was performed for the combined set of instruments for all the exposures (i.e., amino acids and BMI/lifetime smoking) with a threshold of r^2^ < 0.001 within a 10,000 kb window to ensure independence of instruments. MVMR analysis was carried out as described by Sanderson et al.,^40^ where the covariance between genetic associations with each exposure was fixed at zero. Furthermore, we calculated conditional F-statistics to assess the strength of instruments. MVMR analysis was conducted using the MVMR package for R.^43^

#### Instrument strength

To assess instrument strength in MR analysis, we calculated an F statistic for each genetic instrument and I^2^_GX_ statistic for each exposure (i.e., amino acid) in the case of MR-Egger regression.^44^ A high value of I^2^_GX_ (normally greater than 0.9) would be indicative of less than 10% relative bias due to measurement error in the MR-Egger causal estimate, which is equivalent to a scenario of F-statistic greater than 10 in conventional instrumental variable analysis.^44^

#### Relevance of genetic instruments

Our interest in this study is whether maternal circulating amino acids during pregnancy influence fetal growth and hence birthweight. We selected SNPs from a GWAS conducted in the general population (combining women and men) assuming that these SNPs are relevant instruments for our target population (i.e., pregnant women). We assessed the plausibility of this assumption by testing the relevance of the amino acids instruments in predicting maternal circulating amino acids during pregnancy. We were only able to identify one cohort with maternal genotype and circulating amino acids measured during pregnancy (the BiB cohort). In BiB, amino acids were measured as part of an NMR metabolomics analysis, at 24–28 weeks of gestation (further details in^25^). Data were available for 9 of the amino acids and in 2966 women of European ancestry. Amino acids levels were first natural log-transformed, winsorised at 5 SDs and transformed to Z scores, then adjusted for maternal age and top 10 principal components from genomic data. Each of the resulting residuals was regressed against the corresponding SNP (genetic instrument) used in the main MR analysis. A total of 89 SNP-amino acid associations were estimated from this analysis. In addition to comparing GWAS associations to the equivalent in BiB, we also compared them to the same associations in the Fenland study (non-pregnant women). In addition to comparing individual SNP associations between the GWAS, BiB and Fenland, we also compared the meta-analysis estimates of the genetic instrument-amino acid associations for each amino acid across the three different data sources.

### Ethics statement

Ethics approval for BiB has been obtained from the Bradford Research Ethics Committee. Written consent was obtained from all participants. The Fenland study was approved by the National Health Service (NHS) Health Research Authority Research Ethics Committee (NRES Committee–East of England Cambridge Central, ref. 04/Q0108/19), and all participants provided written informed consent. This study only used its summary level data.

### Role of the funding source

The funders had no role in study design, data collection, analysis, or interpretation, or any aspect pertinent to the study.

## Results

### Pair-wise genetic correlations across amino acids

Using data from up to 86,507 individuals, we selected 112 SNPs strongly (*p* < 4.9 × 10^−10^) and independently (r^2^ < 0.05 and ≥ 1 Mb on each side of the sentinel SNP) associated with the blood concentration of at least one of the 20 amino acids (Table S2). Using a list of 110 independent genetic variants after more stringent LD-clumping (r^2^ < 0.01 within a 10,000 kb window), we calculated the pair-wise correlation coefficients for the SNP-amino acid effect estimates based on summary data from previously published GWAS.^27^^,^^28^ We were able to extract 108 SNP-amino acid effect estimates across 8 NMR spectroscopy measured amino acid concentrations (alanine, glutamine, histidine, isoleucine, leucine, phenylalanine, tyrosine and valine) from the GWAS conducted by Kettunen et al.^27^ and 72 SNP-amino acid effect estimates across 7 MS measured amino acids (asparagine, glycine, lysine, methionine, proline, serine and tryptophan) from the GWAS done by Shin et al.^28^

**Fig. 1:**
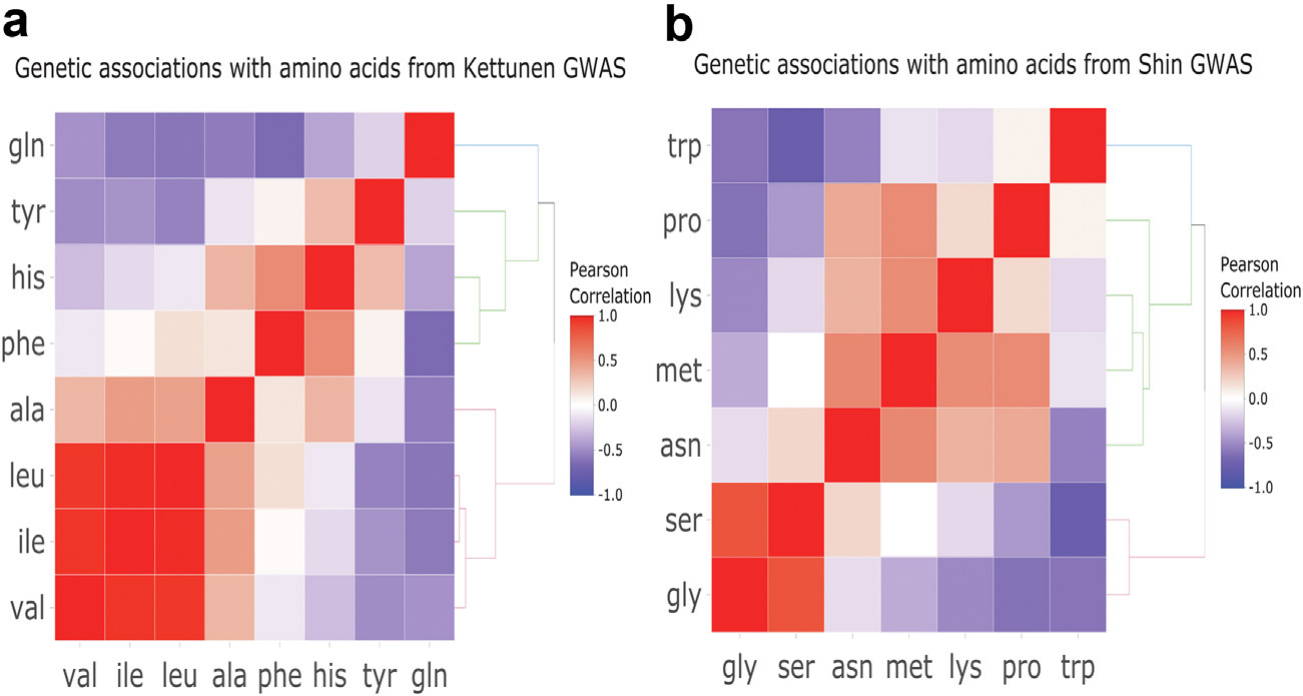
Pair-wise correlations for the SNP-amino acid effect estimates across amino acids measured using Nuclear Magnetic Resonance (NMR) spectroscopy (a) and Mass Spectrometry (MS) (b). Data were extracted from previously published GWAS.^27^^,^^28^ Ala, alanine; asn, asparagine; gln, glutamine; gly, glycine; his, histidine; ile, isoleucine; leu, leucine; lys, lysine; met, methionine; phe, phenylalanine; pro, proline; ser, serine; trp, tryptophan; tyr, tyrosine; val, valine.

We calculated pair-wise genetic correlations between the amino acids on each measurement platform, with the results presented in Fig. 1. Branched chain amino acids (BCAAs; valine, leucine and isoleucine) were highly correlated with one another (r = 0.77–0.91) and serine and glycine were also strongly correlated (r = 0.65). These results point to the clustered nature of genetic regulation of circulating amino acids, which is likely reflecting shared metabolic pathways between amino acids. As an example, valine, leucine and isoleucine are metabolized by a series of reactions catalyzed by the same enzymes to generate intermediates for the citric acid cycle (also known as the tricarboxylic acid cycle or the Krebs cycle) (Fig. S2). Therefore, higher/lower activity of this pathway will affect the concentration of the three branched-chain amino acids. Likewise, serine and glycine are intertwined due to their interconversion as part of one-carbon metabolism, which is essential for nucleotide synthesis and methylation reactions involved in epigenetic regulation and serine is intensively involved in glycine biosynthesis within the glycine metabolism pathway (Fig. S3).

### Genetic variants instrumenting for circulating amino acids

Five of the 110 selected genetic variants were absent from the birthweight GWAS, however we identified proxy SNPs (r^2^ ≥ 0.8) for four of them (no proxy could be found for rs142714816); this led to a total of 106 SNPs remaining as genetic instruments for the 19 amino acids after data harmonization (3 palindromic SNPs were removed and there were no available genetic variants instrumenting for cysteine) (Table S3). These genetic variants explained a proportion of variation in circulating amino acids ranging from 0.13% (glutamate) to 4.76% (asparagine) (Table S4).

### Estimates of causal effects of maternal amino acids on offspring birthweight

#### Main findings

**Fig. 2:**
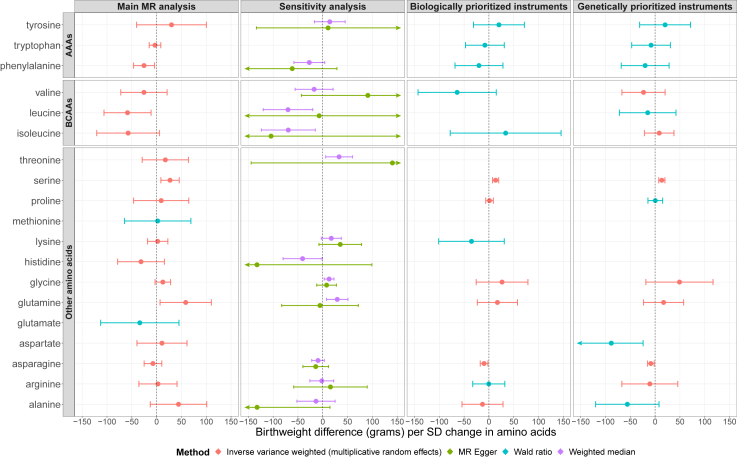
MR estimates of the effects of maternal circulating amino acids on offspring birthweight. In the conservative MR analyses, we chose SNPs that were prioritized in the original metabolites GWAS,^24^ where two approaches (a hypothesis-free genetic approach and a biological knowledge-based approach) were used to prioritize likely causal genes for the observed genetic associations with metabolites. AAAs, aromatic amino acids; BCAAs, branched chain amino acids.

In the main MR analyses, there was evidence suggesting positive causal effects of maternal serine (27 g higher offspring birthweight per SD higher serine, 95% CI: 9, 46) and glutamine (59 g, 95% CI: 7, 110) on offspring birthweight. There was also evidence of inverse causal effects of phenylalanine (−25 g, 95% CI: −47, −4) and leucine (−59 g, 95% CI: −106, −11) on offspring birthweight. There was no strong evidence for causal effects of the remaining amino acids on offspring birthweight (Fig. 2 and Table S5). However, despite using the largest available datasets, some causal effects were imprecisely estimated and, therefore, we cannot discard the presence of biologically meaningful effects for some amino acids, such as alanine (43 g, 95% CI: −14, 100).

#### Sensitivity analyses to explore possible bias due to horizontal pleiotropy

##### Between SNP heterogeneity

If all SNPs instrumenting for a particular amino acid are valid (i.e., without horizontal pleiotropy effects), we would expect the effect estimates to be consistent across SNPs. To assess this, we performed leave-one-out analysis for amino acids having more than one genetic instrument and calculated Cook's distance for amino acids having five or more genetic instruments. Cochrane's Q statistic was used to test between SNP heterogeneity in the causal estimates.

There was evidence of between SNP heterogeneity for some of the amino acids according to the visual inspection of leave-one-out analysis results (Fig. S4), quantification of Cook's distance and Cochrane's Q statistic (Table 1 and Table S6). SNPs in or near several genes (genetically prioritized genes identified in the GWAS conducted by Lotta et al.^24^) that contributed to this heterogeneity were identified (Table 1). Some of these loci, such as *GCKR* (regulating glucokinase) and *CPS1* (catalysing the initial step for the urea cycle, pathway responsible for amino acids degradation and urea synthesis), have been reported to be pleiotropic in previous studies,^45^^,^^46^ whereas other loci, such as *GLS2* and *PPM1K-DT*, have been found to play a key role in the regulation of glutamine and branched-chain amino acids, respectively.

**Table 1:** Influential SNPs detected by Cook's distance and Cochran's Q test results.

Amino acids	Q statistic	Df	I^2^ (%)	*p*-value	Influential SNPs detected by Cook's distance
Alanine	137.73	19	86.20	4.94E-20	rs1260326 (*GCKR*), rs2168101 (*LMO1*)
Asparagine	9.21	4	56.57	0.06	rs12587599 (*ASPG*)
Glutamine	108.51	12	88.94	1.18E-17	rs2657879 (*GLS2*)^a^
Glycine	35.71	12	66.40	3.61E-4	rs715 (*CPS1*)
isoleucine	10.08	4	60.32	3.92E-2	rs1440581 (*PPM1K-DT*)^a^
leucine	8.82	5	43.31	0.12	rs7678928 (*PPM1K-DT*)^a^
lysine	14.52	8	44.90	0.07	rs8056893 (*SLC7A6*)

A cut-off of 4/the number of SNPs was used to detect the influential SNPs by Cook's distance.

Genes mapped to influential SNPs were identified using a hypothesis-free genetic approach in the original cross-platform GWAS of metabolites.^24^

a SNPs included in the below conservative analysis.

##### MR-Egger and weighted median analyses

To explore whether these heterogeneous SNPs were causing bias in our main analyses we undertook MR sensitivity analyses that are more robust to invalid instruments (i.e., weighted median and MR-Egger regression). These require multiple SNP instruments and were conducted for 13 of the 19 amino acids with 5 or more SNPs available as genetic instruments (Fig. 2). Weighted median and MR-Egger regression analysis yielded broadly consistent results with the findings from the main IVW MR analysis (Fig. 2). The one exception was the result for alanine, which in the main IVW analysis had a positive effect on offspring birthweight with wide confidence intervals including the null but in MR-Egger analysis an inverse effect size was revealed though extremely wide confidence intervals also included the null. The MR-Egger intercept also suggested that the IVW result for alanine might be biased by unbalanced horizontal pleiotropy (*p* = 0.02) (Table S7). For all other amino acids for which MR-Egger could be undertaken there was no strong evidence of unbalanced horizontal pleiotropy (all MR-Egger intercept *p*-values >0.05).

##### Conservative MR analysis

In addition to these sensitivity analyses we also explored potential bias due to horizontal pleiotropy by undertaking conservative MR analyses where possible. SNPs mapped to genes directly involved in amino acids metabolism are more likely to be valid instruments for testing the effects of circulating amino acids than SNPs mapped to genes known to be highly pleiotropic (e.g., *GCKR*) or of unknown role in amino acids metabolism. Therefore, we defined conservative sets of SNPs (i.e., SNPs that are more credible instruments for circulating amino acids) and conducted MR analyses (IVW or Wald ratio) based on these. This was done by restricting the genetic instruments to those with established genetic or biological functions indicating direct causal effects on specific amino acids.

Twenty-one SNPs instrumenting for 13 amino acids were selected based on biological plausibility (selection criteria detailed in Methods section), and additional 20 SNPs instrumenting for 14 amino acids were selected based on genetic function (Table S8). As shown in Fig. 2, compared with the findings from the main MR analysis, the conservative analysis produced broadly consistent results. A positive effect of maternal serine on offspring birthweight was confirmed for both biologically and genetically prioritized instruments. Inverse effects of asparagine and aspartate were observed in the conservative analysis, which were not consistently observed in the main MR analysis. In addition, the effect estimates for glutamine and some BCAAs (i.e., leucine and isoleucine) attenuated in the conservative analysis.

##### MVMR analysis with adjusting for maternal BMI and smoking

**Fig. 3:**
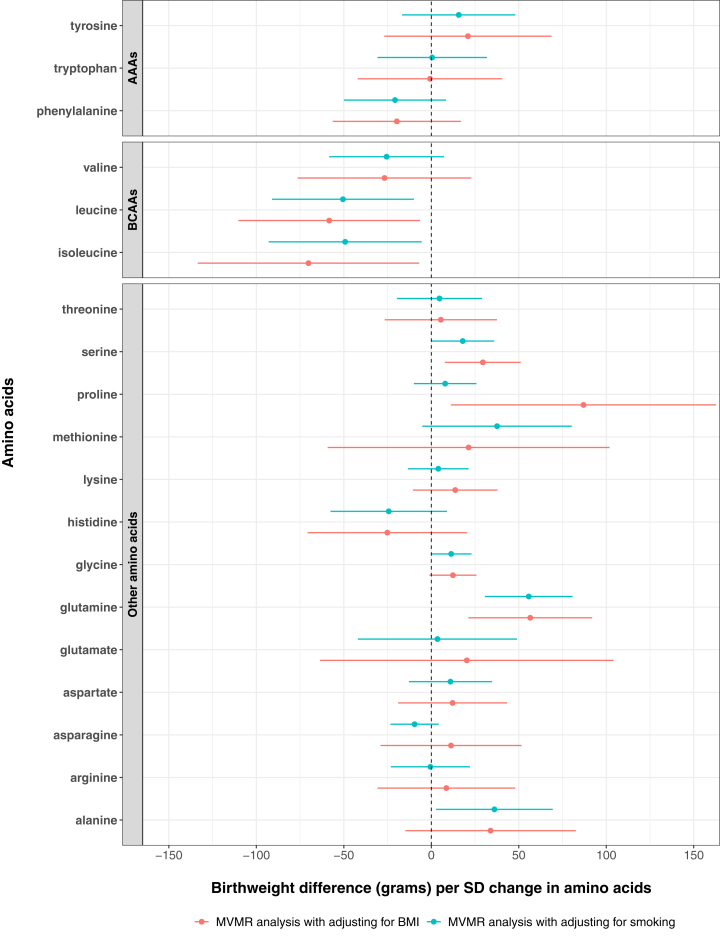
MVMR estimates of the causal effects of maternal circulating amino acids on offspring birthweight with adjusting for maternal BMI and lifetime smoking respectively. AAAs, aromatic amino acids; BCAAs, branched chain amino acids; BMI, body mass index; MVMR, multivariable Mendelian randomisation.

The causal effects of maternal circulating amino acids on birthweight following MVMR adjustment for maternal BMI, and (separately) for lifetime smoking were consistent with the main unadjusted MR analyses, with one exception, suggesting that bias due to horizontal pleiotropy from maternal BMI/smoking was unlikely to explain most of our results (Fig. 3). The one exception was for the potential effect of proline on birthweight. In our main MR (unadjusted) analyses there was no robust evidence of an effect of proline on birthweight (difference in mean birthweight per SD higher proline: 9 g, 95% CI: −46, 65). However, in MVMR analysis adjusting for maternal BMI this increased more than 9-fold to a strong positive effect (87 g, 95% CI: 11, 163). The result from MVMR analysis with adjustment for lifetime smoking (8 g, 95% CI: −10, 26) was consistent with the main unadjusted estimate. Conditional F-statistics ranged from 2.03 (glutamate) to 168.92 (glycine) after adjusting for BMI and from 1.40 (glutamate) to 74.19 (glycine) after adjusting for lifetime smoking (conditional F-statistics for each amino acid can be found in Table S9), suggesting likely weak instrument bias in MVMR analysis.

##### Analyses to explore instrument strength

The instrument strength in the main IVW MR analysis was assessed by F statistic and additionally I^2^_GX_ statistic was calculated to quantify the strength of violation of the ‘NO Measurement Error’ (NOME) assumption, which, if greater than 0.9, should not materially affect the MR-Egger regression estimates.^44^ F statistic ranged from 38.74 to 7504.06 with mean F statistic across SNPs instrumenting for each amino acid ranging from 41 (glutamate) to 633 (glycine). I^2^_GX_ ranged from 0.67 to 0.99 for amino acids where MR-Egger regression analysis was performed (I^2^_GX_ < 0.9 for alanine, histidine, isoleucine, leucine and threonine and I^2^_GX_ > 0.9 for the remaining amino acids) (Table S4 for instrument strength: F statistic and I^2^_GX_), suggesting that MR-Egger analysis estimates for the former 5 amino acids should be interpreted with caution because of potential effect dilution.

##### Testing the genetic instrument relevance to maternal pregnancy amino acids

**Fig. 4:**
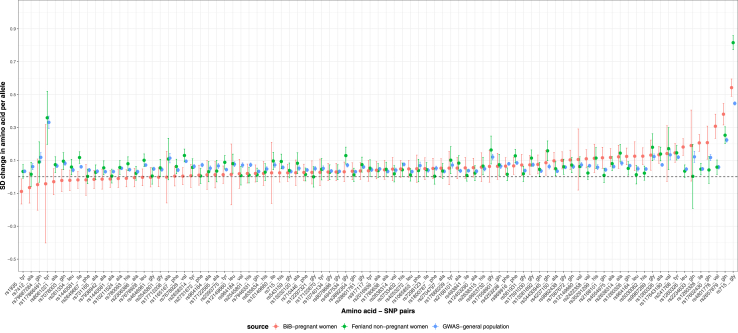
Comparisons of 89 genetic associations with amino acids between the cross-platform metabolites GWAS summary data (women and men), analysis results from the Fenland study (non-pregnant women) and the BiB study (pregnant women).

The SNPs used as genetic instruments for amino acids in this study were obtained from the largest GWAS to date that was done in non-pregnant women and men.^24^ If genetic associations with amino acids differ markedly between women and men, or within women during pregnancy, our MR results could be biased. To explore this, we compared all 89 genetic associations with amino acids from the GWAS to the equivalent associations in a sample of (non-pregnant) women only (N = 4407 Europeans from the Fenland study) and also a cohort of women (N = 2966) with amino acids measures at 26–28 weeks gestation (the BiB cohort). A total of 67 of these genetic associations were consistent across all three samples (Fig. 4; heterogeneity *p* > 0.05). For the remaining 22 associations there was some evidence of heterogeneity (*p* ranging from 3.72E-62 to 0.04). One reason for the heterogeneity might be low imputation quality of some SNPs in BiB study, such as rs4801776 (imputation quality score INFO = 0.54), rs1935 (INFO = 0.65) and rs2168101 (INFO = 0.69), and these SNP-amino acid pairs were depicted as extremely heterogenous in Fig. 4. Another reason for the heterogeneity is the presence of sex-specific effect between specific variants and amino acids. For example, it has been reported that there are substantial sex differences in the effect size of the variant rs715 on glycine (genetic association has higher magnitude for women than men),^46^ and in our study it was confirmed by the magnitude difference between BiB pregnant women, Fenland non-pregnant women and GWAS general population (both pregnant and non-pregnant women higher than general population) as shown in Fig. 4. After accounting for the above potential reasons, the genetic associations with amino acids across the three data sources were broadly consistent, which provides evidence of using top hits from GWAS summary data as genetic instruments of maternal pregnancy circulating amino acids in this study. The consistency was confirmed in the comparison of the meta-analysis estimates of the genetic instrument–exposure associations across the three different data sources except for glycine, phenylalanine where substantial heterogeneity between estimates in the Fenland non-pregnant women and GWAS results in the general population was observed (Fig. S5).

## Discussion

In the present study, we explored the causal relationships between 19 maternal circulating amino acids and offspring birthweight using two-sample MR analysis and data from recent metabolites and birthweight GWAS, which included up to 86,507 and 406,063 participants, respectively. Our results are supportive of maternal circulating glutamine and serine having positive, and leucine and phenylalanine having negative effects on offspring birthweight. These findings are broadly supported by a series of sensitivity analyses exploring bias due to potential violation of MR assumptions. Despite using the largest GWAS to date for amino acids and birthweight, it should be noted that, for some amino acids, estimates were imprecise and key sensitivity analyses could not be conducted due to the low number of selected SNPs or lack of selected SNPs mapping to genes directly involved in amino acids metabolism.

Glutamine and glutamate are non-essential amino acids that become conditionally essential amino acids in catabolic stress states, which include pregnancy as fetal demand exceeds maternal synthesis.^47^ Our conservative MR analyses and review of the literature suggest that the positive effect of maternal glutamine on offspring birthweight might be isoenzyme dependent.^48^^,^^49^ Specifically, when glutamine was instrumented by the missense variant rs2657879 in *GLS2* there was evidence of a strong positive effect, whereas when instrumented by rs7587672, which is an eQTL for *GLS*, there was an imprecise inverse effect (Fig. S6A). As *GLS2* encodes the enzyme which catalyzes the conversion of glutamine to glutamate and ammonia and is primarily expressed in the liver (i.e., liver-type isozyme) and *GLS* encodes the kidney-type isozyme,^50^ we postulate that an overall positive causal effect of circulating glutamine level during pregnancy on offspring birthweight might be primarily driven by liver-type isoenzyme mechanism. This is indirectly supported by the positive association driven by rs17602430 (*SLC38A2*) (Fig. S6B) that is sodium-dependent neutral amino acids (including glutamine) transporter in system A.^51^

During late pregnancy glycine plays a critical role in fetal growth because it is a primary source of one-carbon necessary for both synthesis and methylation of DNA and other molecules.^52^ However, there is evidence that glycine is relatively poorly transported across the human placenta and placental glycine supply is thought to be lower than fetal demand.^53^ It has been hypothesized, and with some support from sheep and human pregnancy tracer studies, that maternal circulating serine is not transported to the fetal circulation via the placenta but is used within the utero-placental tissues to synthesize glycine, and via this mechanism makes an important contribution to fetal glycine supply.^54^^,^^55^ Our findings appear to support this hypothesis, that is, a causal role of maternal circulating serine on offspring birthweight was found but little evidence on causal effects of maternal circulating glycine on offspring birthweight. For glycine, results from conservative analysis (Fig. S7) based on two SNPs, rs17591030 (*GLDC*) and rs9923732 (*GCSH*), did not support a causal effect of it, though results based rs561931 (*PHGDH*) and rs4947534 (*PSPH*) suggested positive effects. The reason for this may be the distinct biological pathways involved. The *GLDC* and *GCSH* loci are involved in glycine degradation, whereas *PHGDH* and *PSPH*, encode enzymes involved in the de novo biosynthesis of serine.^46^ Given the interlinked metabolism of serine and glycine, this might explain the observed potential positive effects of glycine on birthweight when estimated using *PHGDH* and *PSPH* compared to *GLDC* and *GCSH*. Further exploration of this, for example, using multivariable MR might be valuable but would require large sample sizes.

Unlike glutamine, serine and glycine, which are non-essential amino acids and use sodium-dependent A system for placental transport, BCAAs, including valine, leucine and isoleucine, use sodium-independent L system and cross the placenta more rapidly.^56^ Previous studies have reported that maternal higher concentrations of essential amino acids, including BCAAs, were associated with higher risk of intrauterine growth restricted pregnancies.^7^^,^^57^ In the present study, an inverse effect of maternal circulating leucine on offspring birthweight was found in the main MR analysis, which was directionally consistent with estimates from sensitivity analyses (although effect estimates were attenuated). Leucine has been proposed as a modulator of fetal muscle protein synthesis through the activation of the intracellular mammalian target of rapamycin (mTOR) signaling pathway.^58^, ^59^, ^60^, ^61^ Besides, in previous MR studies, higher circulating BCAA has been related to higher risk of type 2 diabetes^62^ and elevated maternal blood glucose has been confirmed to increase offspring birthweight.^17^ Thus, a positive association between BCAA levels and offspring birthweight would be expected but interestingly the findings in this study provided evidence of an opposite relationship, which was also observed in a recent metabolomic study.^63^ Given the complex biological mechanisms involved in the BCAA metabolism and its close link with insulin resistance,^64^^,^^65^ further MR studies in large numbers of pregnancies to dissect the impact of maternal fasting insulin and circulating BCAAs on offspring birthweight are warranted.

For phenylalanine, our main findings suggested an inverse effect on offspring birthweight, which was supported by similar, although imprecise, effect estimates across different methods of sensitivity analyses. There is evidence, from one small observational study of 20 twins, suggesting a marked reduction in the fetal circulating concentrations of amino acids including phenylalanine transported by system L in small for gestational age twins compared to appropriate for gestational age twins, though no differences in maternal amino acids concentrations were observed between these two groups,^66^ and we did not identify other studies in larger samples of the same association. Further research is required to better understand the potential mechanisms underlying our findings.

The main strengths of our study include the utilization of maternal genetic effects from a large birthweight GWAS (total sample size up to 406,063 individuals) accounting for fetal genetic effects, in a two-sample MR framework to improve causal inference. Additionally, we undertook a series of sensitivity analyses to explore bias due to violation of MR assumptions. We also used the largest and most comprehensive amino acid GWAS to select genetic instruments with validation in a sample of pregnant women. We were able to demonstrate consistent associations between the GWAS and a sample of women only, and also with an independent cohort of pregnant women, with the exception of one SNP that had previously been identified as female specific.^46^ We accounted for possible population stratification by restricting to European ancestry and using GWAS summary data accounting for population structure (e.g., via principal components of ancestry or using mixed models). Our conservative analysis, conducted by restricting the genetic instruments to those with established genetic or biological functions in amino acids metabolism, minimizes the potential for bias due to horizontal pleiotropy arising from the use of SNPs that do not have specific effects on amino acids metabolism pathways. In addition, we used MVMR to explore specific bias due to horizontal pleiotropy via maternal BMI and lifetime smoking. For the vast majority of results, we found no evidence of this. The strong positive association of proline with birthweight in MVMR analysis after adjusting for maternal BMI we believe to be an artefact related to not having the SNP or a suitable proxy for rs3970551 as one of the proline instruments. This variant was one that we had a priori was identified as a genetically and biologically functional variant for proline, meaning that the strong adjusted result after including all the other variants but not this one and the accentuated effect is likely due to this. Taking these MVMR results together with our other sensitivity analyses, bias due to horizontal pleiotropy is unlikely to have notably biased our findings.

One of the limitations of our study is in one major component of the birthweight GWAS data, that is UK Biobank study, which accounts for 80% of the sample size in the birthweight GWAS, birthweight was retrospectively reported by mothers.^30^ Additionally, as UK Biobank had a low response rate (∼5%) at recruitment, potential selection bias could be an issue in the genetic association studies and consequent MR analyses.^67^ Last, our study focussed on maternal genetic variants regulating amino acids concentration in maternal circulation and the potential consequences for fetal growth; however, the impact of amino acids on fetal growth is likely to result from a complex interplay of maternal, fetal and placental mechanisms, which should be explored in future studies. As an example, there is emerging evidence that maternal metabolism affects maternal circulating amino acids but also fetal amino acids uptake by regulating placental transport mechanisms,^68^, ^69^, ^70^ and therefore our results should be interpreted with caution.

In conclusion, our findings indicate that maternal genetically predicted levels of glutamine and serine increase offspring birthweight, and leucine and phenylalanine appear to reduce offspring birthweight. Despite using the largest GWAS, several causal effects were imprecisely estimated, including some that might indicate potentially important clinical effects, such as for alanine. Thus, larger GWAS of amino acids and birthweight in particular are needed to replicate our findings and elucidate mechanisms by which these amino acids could influence fetal growth. In addition, the mechanisms underlying these effects are likely to involve how amino acids are transported across the placenta and the role of fetal responses and their genotypes in influencing placental transmission. Further research is required to gain insights into those mechanisms. Large-scale, high-quality randomized controlled trials are key to establish whether intervening on maternal circulating amino acids during pregnancy (e.g., via supplementation) can be a useful strategy to optimize healthy fetal growth.

## Contributors

All authors read and approved the final version of the manuscript. DAL and MCB developed the idea for the project, J Zhao, DAL, MCB designed the study and analysis plan, J Zhao undertook all analyses, IDS, CL provided additional amino acid summary data that was not in the public domain at the time of starting analyses, DAL, DM, JW obtained and managed genomic and phenotypic data collection in Born in Bradford. J Zhao wrote the first draft of the paper with input from DAL and MCB and all authors contributed to interpretation of results and critical revision of drafts. The following authors have directly accessed and verified the underlying data, J Zhao and MCB.

## Data sharing statement

Data on birthweight have been contributed by the EGG Consortium using the UK Biobank resource and are available at www.egg-consortium.org. All genome-wide summary statistics for amino acids are available at https://omicscience.org/apps/crossplatform/. The data in BiB are fully available, via a system of managed open access, to any researchers. Full information on how to access BiB data can be found at https://borninbradford.nhs.uk/research/how-to-access-data/.

## Declaration of interests

DAL has received support from 10.13039/100004374Medtronic Ltd and 10.13039/100016545Roche Diagnostics for research unrelated to this study. All other authors declare no conflicts of interest.

## Appendix B Supplementary data

Supplementary Tables_Dec2022.xlsx
