# Supplementary material for: Causal effects of maternal circulating amino acids on offspring birthweight: a Mendelian randomisation study
Source: eBioMedicine. 2023 Jan 23;88:104441. doi: 10.1016/j.ebiom.2023.104441 (PMC9879767; doi:10.1016/j.ebiom.2023.104441)
Supplement: Supplementary Figures [file mmc2.docx]

Supplementary Figures

Figure S1. Overlap in SNPs selected as genetic instruments for amino acids.

Figure S2. Major metabolic pathways for branched chain amino acids degradation.

Figure S3. Major metabolic pathways for serine and glycine biosynthesis.

Figure S4. Leave-one-out analysis result.

Figure S5. Comparison of the meta-analysis estimates of the genetic instrument-exposure associations across the three different data sources (BiB study, Fenland study and metabolites GWAS in the general population).

Figure S6. Comparison between the conservative MR analysis and main MR analysis for glutamine.

Figure S7. Comparison between the conservative MR analysis and main MR analysis for glycine.


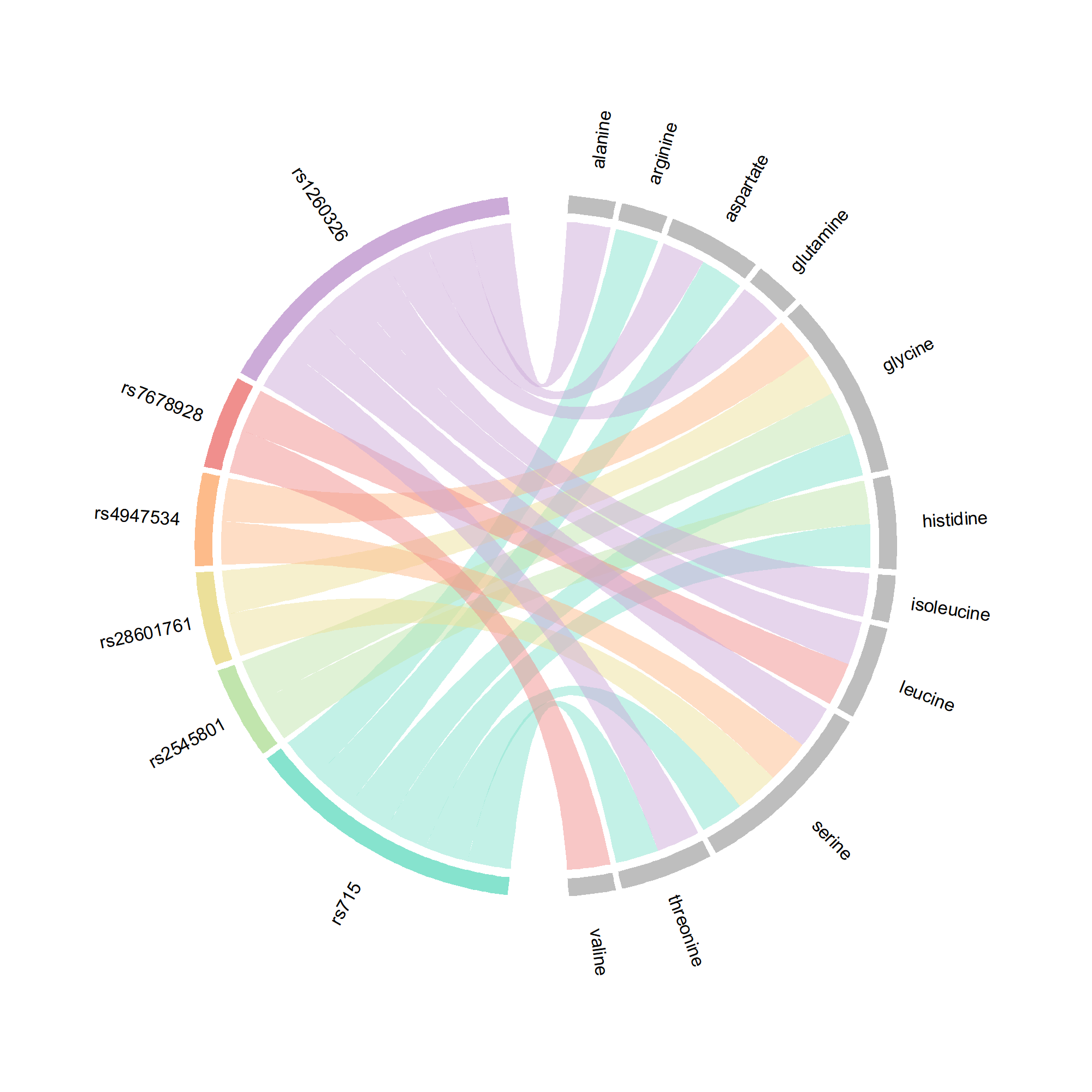


Figure S1. Overlap in SNPs selected as genetic instruments for amino acids.


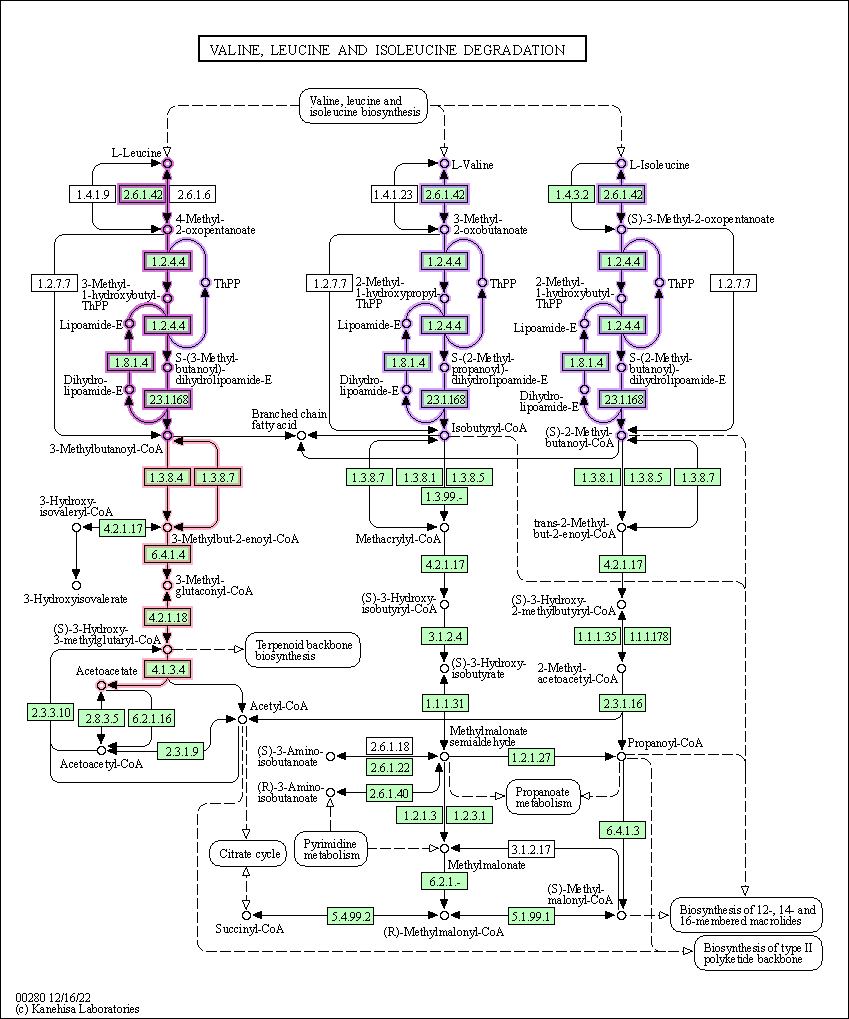


Figure S2. Major metabolic pathways for branched chain amino acids degradation (downloaded from KEGG Pathway Map: <https://www.genome.jp/pathway/hsa00280+N00832> with copyright permission granted).

References:

Kanehisa, M. and Goto, S.; KEGG: Kyoto Encyclopedia of Genes and Genomes. Nucleic Acids Res. 28, 27-30 (2000).


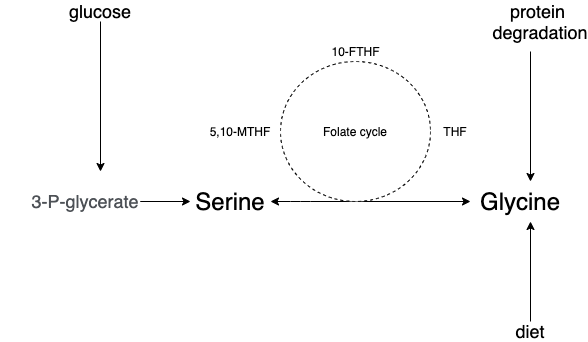


Figure S3. Major metabolic pathways for serine and glycine biosynthesis.

THF: tetrahydrofolate, 5,10-MTHF: N⁵-N¹⁰-methylenetetrahydrofolate, 10-FTHF: 10-formyltetrahydrofolate

Note: This is a hand-drawn pathway figure based on our knowledge for illustration purpose.

Figure S4. Leave-one-out analysis result.


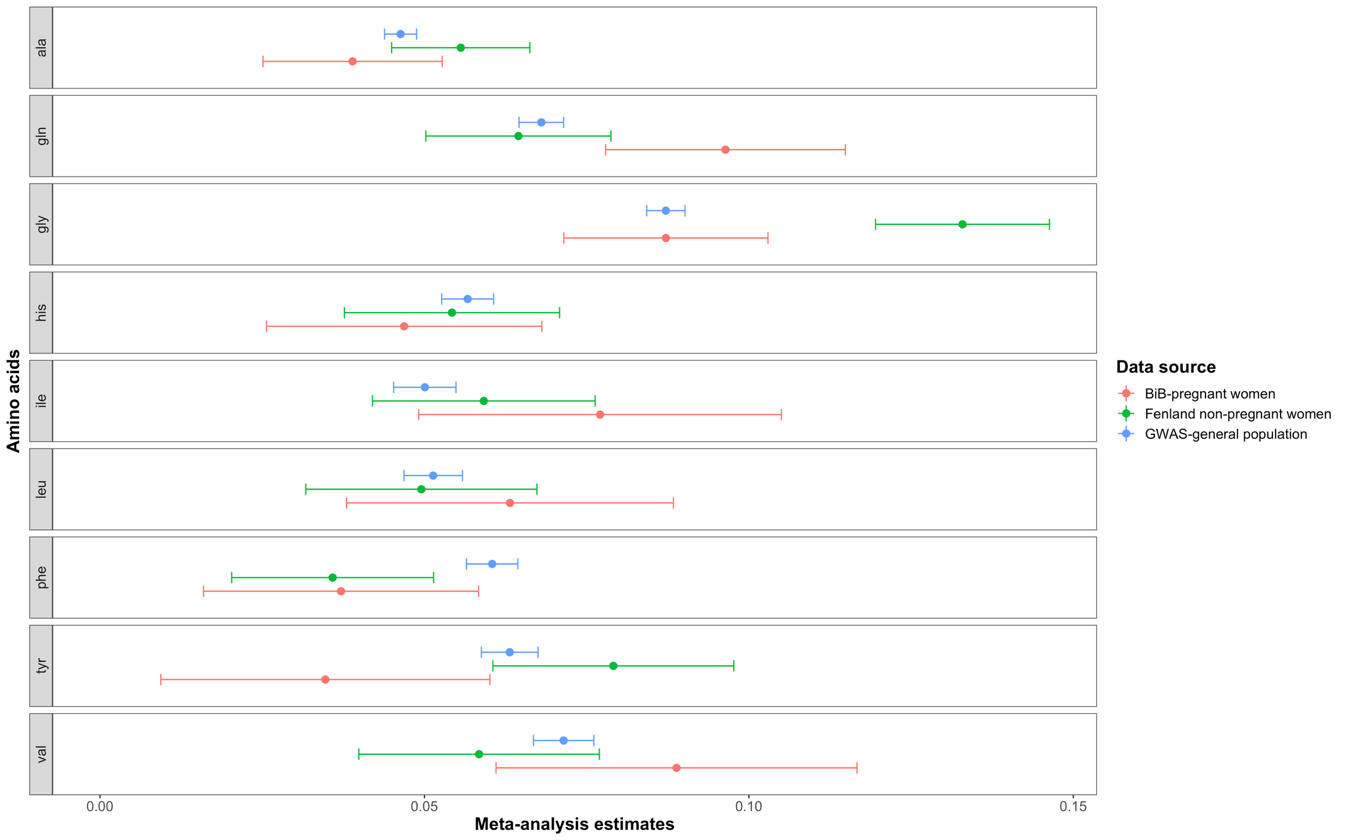


Figure S5. Comparison of the meta-analysis estimates of the genetic instrument-exposure associations across the three different data sources (BiB study, Fenland study and metabolites GWAS in the general population).

Figure S6. Comparison between the conservative MR analysis and main MR analysis for glutamine.

Figure S7. Comparison between the conservative MR analysis and main MR analysis for glycine.
